# Species Difference of CD137 Ligand Signaling in Human and Murine Monocytes

**DOI:** 10.1371/journal.pone.0016129

**Published:** 2011-01-14

**Authors:** Qianqiao Tang, Dongsheng Jiang, Zhe Shao, Julia M. Martínez Gómez, Herbert Schwarz

**Affiliations:** 1 Department of Physiology, and Immunology Programme, Yong Loo Lin School of Medicine, National University of Singapore, Singapore, Singapore; 2 NUS Graduate School for Integrative Sciences and Engineering, National University of Singapore, Singapore, Singapore; Universidade Federal do Rio de Janeiro, Brazil

## Abstract

**Background:**

Stimulation of CD137 ligand on human monocytes has been shown to induce DC differentiation, and these CD137L-DCs are more potent than classical DCs, in stimulating T cell responses in vitro. To allow an in vivo evaluation of the potency of CD137L-DCs in murine models we aimed at generating murine CD137L-DCs.

**Methodology/Principal Findings:**

When stimulated through CD137 ligand murine monocytes responded just as human monocytes with an increased adherence, morphological changes, proliferation and an increase in viable cell numbers. But CD137 ligand signaling did not induce expression of inflammatory cytokines and costimulatory molecules in murine monocytes and these cells had no T cell stimulatory activity. Murine monocytes did not differentiate to inflammatory DCs upon CD137 ligand signaling. Furthermore, while CD137 ligand signaling induces maturation of human immature classical DCs it failed to do so with murine immature classical DCs.

**Conclusions/Significance:**

These data demonstrate that both human and murine monocytes become activated by CD137 ligand signaling but only human and not murine monocytes differentiate to inflammatory DCs.

## Introduction

Mice have long been invaluable in helping immunologists to understand how immunity works, especially when systemic effects of immune modulations were studied. Many immunotherapeutic approaches, such as neutralization of inflammatory cytokines for treatment of autoimmune disease have been pioneered in mice [1].

Despite many similarities, differences exist between the human and murine immune system. For example peripheral monocytes in man consist of two subpopulations of which the CD14^high^, CD16^−^ cells constitute to 90–95% and the CD14^dim^, CD16^+^ cells constitute 5–10% [2]. CD14 and CD16 are not suitable to distinguish murine monocyte subpopulations. Rather, in the murine system monocyte subpopulations are classified as CD115^+^, Ly6C^high^ and CD115^+^, Ly6C^low^, respectively, which constitute 50% each [3].

Both human and murine monocytes express CD137 ligand (TNFSF9, 4-1BB ligand), a member of the TNF superfamily [4], [5]. CD137 ligand not only sends signals to CD137-expressing cells but it is a transmembrane protein on the cell surface that can also deliver signals into the cells it is expressed on (reverse signaling), [6]. Peripheral human monocytes are activated by CD137 ligand signaling, evidenced by enhanced adherence, increased expression of ICAM-1 and secretion of proinflammatory cytokines [7], [8], increased survival [9], induction of proliferation [10], [11] and enhanced migration [12], [13]. Murine macrophages are similarly activated by CD137 ligand signaling also leading to enhanced adherence, increased expression of ICAM-1 and secretion proinflammatory cytokines [14], [15].

CD137 ligand signaling can also induce maturation of human immature monocyte-derived DCs leading to an enhanced expression of costimulatory molecules, IL-12 secretion, and an enhanced capacity of the DCs to stimulate T cell proliferation, IFN-γ secretion and in vivo migration towards a CCL19 gradient [16]–[18].

Two recent studies report that CD137 ligand signaling induces full human monocyte to DC differentiation. CD137 ligand signaling triggered by a monoclonal anti-CD137 ligand antibody and complemented by IL-4 induced costimulatory molecule expression and T cell stimulatory activity [19]. However, recombinant CD137 protein as a sole factor is sufficient to induce human monocyte to DC differentiation and these CD137L-DCs are more potent than classical DCs in inducing proliferation, IFN-γ secretion and perforin expression by T cells [20].

These data indicate that CD137L-DCs may also be more potent in inducing protective T cell responses than classical DCs. However, a reliable conclusion about the potency of the different DC populations should be based on in vivo experiments. As these would be most easily performed in mice we tested whether CD137 ligand signaling also induces DC differentiation in murine monocytes, so that murine CD137L-DCs can be tested for their ability to induce anti-pathogen and anti-tumor immune responses in vivo.

Just as in human monocytes CD137 ligand signaling induced attachment, morphological changes and proliferation in murine monocytes. However, neither monocyte to DC differentiation nor maturation of immature DCs was induced in the murine system pointing to a species difference in the effects of CD137 ligand signaling between human and murine monocytes.

## Materials and Methods

### Mice

Female Balb/C mice between 6 and 8 weeks of age were used as a source of bone marrow cells. Animals were specific pathogen free, and kept with free access to food and water in the animal care facility at the National University of Singapore under the institutional guidelines for usage of experimental animals (protocol number 018-10).

### Isolation of CD11b^+^, Ly6G^−^ monocytes from bone marrow

The femur bones of Balb/C mice were dissected and the bone marrow was flushed out aseptically with phosphate-buffered saline (PBS), 2 mM EDTA by using a 10 ml syringe and 27G needle. Total bone marrow cells were passed through 30((m filter (Miltenyi Biotec, Bergisch Gladbach, Germany), washed with PBS, 2 mM EDTA and resuspended in RPMI1640 (Sigma, St. Louis, MO, USA), 10% fetal bovine serum (FBS).

The CD11b^+^, Ly6G^−^ monocytes were isolated by negative selection using the mouse monocyte enrichment kit (Stemcell Technologies, Vancouver, CA) following the manufacturer's instructions. Briefly, fresh bone marrow cells were labeled with a cocktail of biotinylated antibodies against a panel of antigens expressed on T, B, NK, DCs, progenitor cells and granulocytes, followed by anti-biotin microbeads. The cell suspension was incubated within a 5 ml polystyrene tube that fits in the Easysep@ magnet device. Unlabled monocytes were obtained by inverting the tube in the magnet and dispensing the cell solution into a new tube.

### Recombinant proteins and chemicals

Recombinant human CD137-Fc protein was purified from supernatants of stable transfected CHO cells by protein G sepharose, as described previously [21]. The endotoxin concentration in the CD137-Fc protein is 55 I.U./mg. Human IgG1 Fc protein was purchased from Accurate Chemical and Scientific Corporation (Westbury, NY, USA). Recombinant mouse CD137-Fc protein was purchased from R&D systems (Minneapolis, MN, USA). Recombinant murine TNFRI-Fc and CD134-Fc protein were obtained from R&D Systems. Recombinant murine GM-CSF and IL-4 were purchased from PeproTech (Rocky Hill, NJ, USA). LPS was obtained from Sigma.

### Antibodies and flow cytometry

Phycoerythrin (PE)-conjugated and Flourescein isothiocyanate (FITC) labeled rat anti-mouse mouse CD11c, CD14, CD80, CD86, F4/80, major histocompatibility complex (MHC) class II, and respective isotype controls (rat IgG2a, rat IgG2b, Armenian Hamster IgG) were purchased from eBioscience (San Diego, USA).

Flow cytometry was performed on a Cyan flow cytometer (Dako, Denmark) with Summit software. Nonspecific staining was controlled by isotype-matched antibodies.

### Cell count by flow cytometry

For the flow cytometry based cell count, cells were harvested after incubation with 10 mM EDTA for 10 min. Cells were centrifuged and resuspended in PBS and stained with 0.5((g/ml 7-AAD for 15 min at RT in dark. After washing three times the cells were resuspended in 450((l PBS. 50((l of Sphero Accucount Blank Particles (1000 beads per (l) were added to the suspension and mixed. Samples were analyzed by flow cytometry with CyanTM (Beckman Coutler, CA, USA). 7-AAD-negative cells were gated as live cells. Beads and target cells were gated separately on the forward/side scatter plot based on the different sizes. The number of cells in each sample was calculated by the following formula: Number of cells in sample  =  (50,000 x Number of counted cells)/Number of counted beads.

### Proliferation assay

Cell proliferation was determined by ^3^H-thymidine incorporation. Cells were pulsed with 0.5 µCi of ^3^H-thymidine (PerkinElmer, Boston, MA) for the last 24 h of the culture period. The cells were then harvested onto a Packard Unifilter Plate using a MicroMate 196 Cell Harvester and counted using a TopCount Microplate Scintillation Counter (Packard Instruments, Meriden, CT).

### Phagocytosis Assay

Yellow-green flourescent carboxylated-modified microspheres (FluroSpheres, Molecular Probes) were added at a ratio of 50 beads per cell at 37°C in the dark. Cells undergoing the same treatment but without the additions of beads served as negative controls. After 1 h incubation, the reaction was arrested by addition of 1 ml ice-cold PBS. Cells were then washed in PBS and trypsin-EDTA was added to dislodge surface adherent beads. Cells were washed again and resuspended in FACS buffer for flow cytometry analysis.

### Allogeneic mixed lymphocyte reaction

2×10^5^ CD11b^+^, Ly6G^−^ monocytes per well were cultured in 24-well plates that had been precoated with 10 µg/ml of Fc or CD137-Fc. In parallel, cells were treated with 100 ng/ml GM-CSF +25 ng/ml IL-4 (Peprotech) to generate immature classical DCs. Maturation of DCs and CD137-treated monocytes was induced on day 6 by addition of 1 µg/ml LPS (Sigma). Subsequently, cells were harvested by incubation with 10 mM EDTA at RT for 10 min and washed twice with PBS, and served as stimulator cells. T cells were isolated from splenocytes of 6-week old C57/BL6 mice by magnetic selection using the Pan T cell isolation kit (Miltenyi Biotec). 10^5^ T cells were cocultured with stimulator cells at a 10∶1 ratio in 96-well round bottom plates, and proliferation was determined on day 3 by ^3^H-thymidine incorporation.

### ELISA

The concentrations of IL-10, IL-12p70 and soluble CD137 in cell supernatants were determined by mouse IL-10, IL-12 (p70), murine soluble CD137 DuoSet (R&D) and MCP-1 (Peprotech) according to the manufacturers' instructions. All measurements were done in triplicate.

### Microscopy

Morphological changes of cells were documented by using a Zeiss Axiovert 40 inverted microscope (Zeiss, Göttingen, Germany) and Canon PowerShot G6 digital camera.

### Statistics

Statistical significance was determined by the two-tailed unpaired Student's t-test.

## Results

### CD137 ligand signaling induces activation of murine monocytes

As a source for murine monocytes CD11b^+^, Ly6G^−^ bone marrow cells were employed. CD137 ligand signaling was initiated as previously described for human peripheral monocytes. Cells were grown on plates that had been coated with a fusion protein consisting of the extracellular domain of human or murine CD137 fused to the constant domain of human IgG1 (Fc). Wells coated with an equal concentration of the Fc protein were used as negative controls.

**Figure 1:**
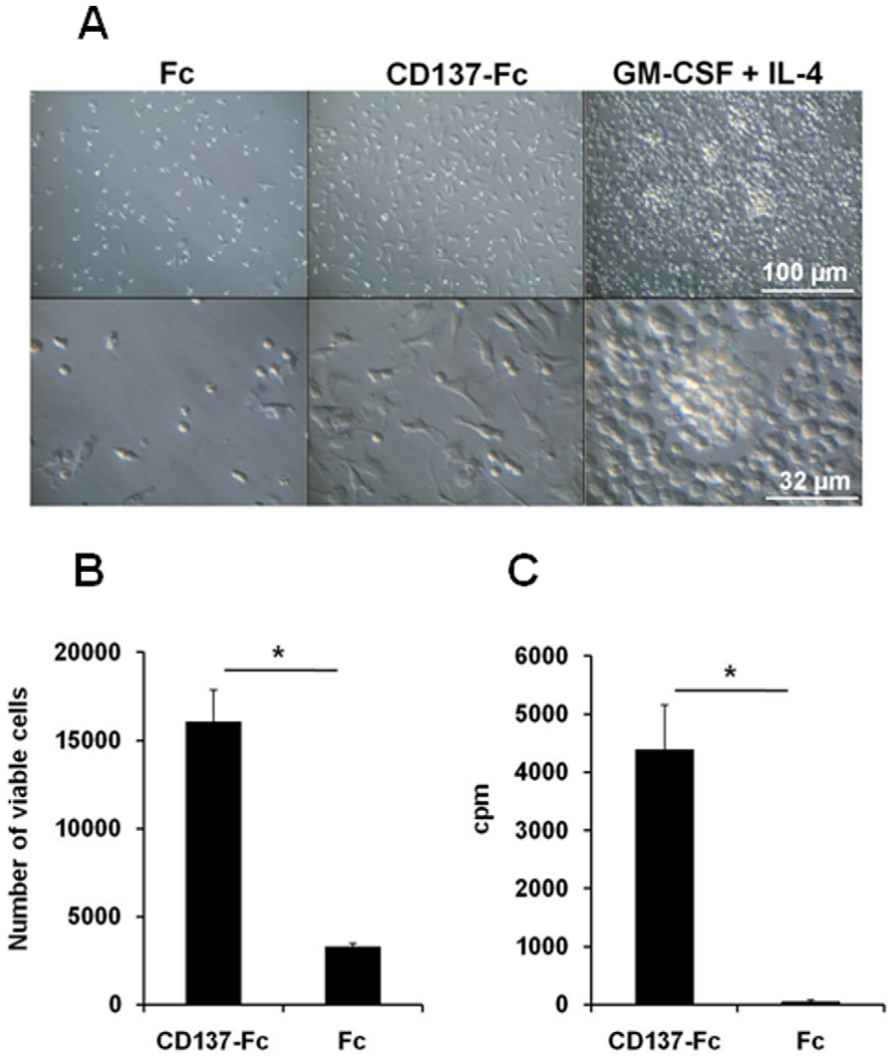
Activation of murine monocytes by recombinant CD137 protein. Monocytes were cultured under indicated conditions for 7 days. (A) Morphological changes were documented by photography at 20x (upper panel) and 63x (lower panel) magnifications. (B) Numbers of viable cells were determined by flow cytometry using Sphero Accucount Blank Particles. (C) Proliferation was determined by ^3^H-thymidine incorporation at 3 day of culture. *: p<0.05. Data are representative of three independent experiments.

In response to CD137 ligand signaling the cells attached to the plates, spread and formed lamellipodia (Figure 1A), similar to what has been shown for human monocytes [7]. In contrast, classical DCs induced by GM-CSF + IL-4 showed formed clusters and a substantial proportion of the cells remained in suspension. Of the Fc-treated control monocytes only few cells attached to the plate, and majority of the attached cells lacked lamellipodia formation.

After a 7 day culture period five-times more live cells were present in the CD137-Fc-coated wells compared to Fc-coated wells (Figure 1B). The increased number of live cells resulting from treatment with CD137-Fc protein could be the result of prolonged cell survival and/or induction of proliferation. Indeed, CD137 ligand signaling induced a significant proliferation of monocytes while control cultures did not proliferate (Figure 1C). These data demonstrate that in murine and human monocytes CD137 ligand signaling induces adherence, morphological changes and proliferation in a comparable way.

### Absence of proinflammatory activation markers

**Figure 2:**
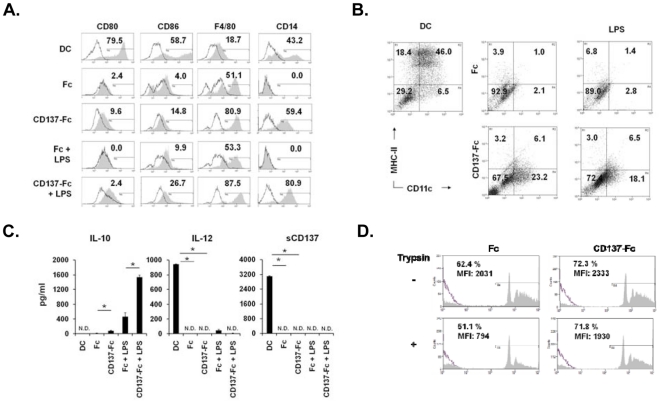
Absence of DC characteristics. Monocytes were cultured under indicated conditions for 7 days. (A) Expression of CD80, CD86, F4/80 and CD14 were determined by flow cytometry. Numbers in the graphs represent the percentages of positively stained cells (grey histograms) compared to the isotype control (open histograms). (B) Expression of CD11c and MHC-II were determined by flow cytometry. Numbers in the graphs represent percentage of the population in each quadrant. (C) Cytokine secretion. The concentrations of (A) IL-10, (B) IL-12, and (C) soluble CD137 in the supernatants were measured by ELISA. *p<0.05. N.D: Not detectable. (D) Phagocytosis was determined by adding flourescent beads to cells at a ratio of 50∶1. The flourescence was determined by flow cytometry. Numbers in the graphs indicate the percentages of positive cells and mean fluorescence intensities (MFI). Half of the harvested cells were trypsinized to remove non-phagozytosed beads that might have stuck on the surface of the cells. Data are representative of three independent experiments.

In human monocytes CD137 ligand signaling induces strong expression of costimulatory molecules which is further enhanced by DC maturation signals such as LPS and IFN-γ [18]–[20]. However, in murine monocytes CD137 ligand signaling only marginally induced expression of CD80 and CD86 and the addition of LPS had little or no effect, while high levels were expressed on classical DCs (Figure 2A). The reverse was the case for F4/80, a murine macrophage marker which was low on classical DCs and high on CD137-treated monocytes (Figure 2A). CD14 was expressed by both classical DCs and CD137-Fc-treated monocytes but not by Fc-treated control monocytes (Figure 2A).

Expression of CD11c, a murine DC marker was substantially enhanced on CD137-treated monocytes (about 10-fold, from 3.1 to 29.3%) but few of these CD11c^+^ cells expressed also MHC class II and only low levels. As in the case of costimulatory molecule expression the addition of LPS had no effect. In contrast, among classical DCs the CD11c^+^, MHC class II^+^ fraction was the largest subpopulation (46%), and expressed high levels of MHC class II (Figure 2B).

IL-12 is secreted by mature DCs and is involved in mediating T cell stimulation, while IL-10 is a potent inhibitor of DC activities. As expected classical mature DCs secreted IL-12 but not IL-10 (Figure 2C). The pattern was the opposite for CD137-Fc-treated monocytes which produced IL-10 but not IL-12. The Fc-treated control cells also produced IL-10 and no IL-12. While stimulation by LPS had little effect on expression of CD80, CD86, MHC class II and IL-12, it synergized with CD137 ligand signaling in inducing IL-10 secretion (Figure 2C).

Soluble forms of CD137 (sCD137) are generated by differential splicing and may limit DC activation [22], [23]. Murine splenic DCs and bone marrow-derived DCs have been reported to secrete sCD137, and maturation by LPS enhances sCD137 secretion [24]. We could confirm high sCD137 secretion by classical mature bone marrow-derived DCs but found that CD137-Fc-treated murine monocytes did not secrete sCD137 (Figure 2C). This is in line with the missing IL-12 secretion, expression of costimulatory molecules and the T cell stimulatory activity of CD137-Fc-treated murine monocytes.

Phagocytosis is reduced during differentiation of human monocytes to classical DCs and CD137L-DCs [20], [25]. In contrast, CD137 ligand signaling increased phagocytosis of murine monocytes (Figure 2D). This difference became even more pronounced when beads attached unspecifically to the cell surface were removed by trypsinization of the cells (Figure 2D).

The failure of CD137 ligand signaling to induce expression of costimulatory molecules and proinflammatory cytokines in murine monocytes contrasts its activities in human monocytes. Also, in human monocytes CD137 ligand signaling reduced IL-10 levels and the phagocytotic capacity while in murine monocytes it had the opposite effect. These data indicate (1) that there is a species difference in the response of human and murine monocytes to CD137 ligand signaling, and (2) that murine monocytes stimulated by CD137 protein may not become inflammatory DCs.

### CD137 ligand signaling fails to induce DC differentiation and maturation

**Figure 3:**
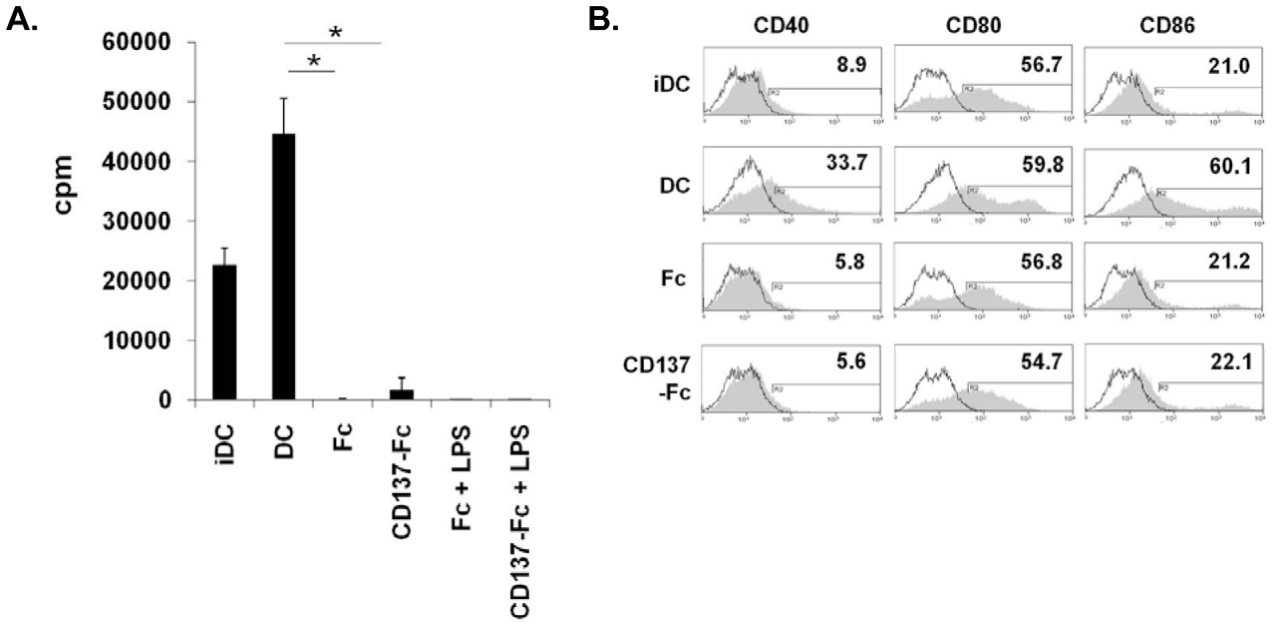
CD137-treated monocytes exert no DC activity. (A) Monocytes from Balb/C mice were cultured for 7 days on plates with immobilized Fc or CD137-Fc protein and where indicated stimulated by LPS for the last 24 h. The cells were cocultured with T cells from C57/Bl6 mice in an allogeneic MLR at a ratio of 1∶10. The rate of proliferation was determined by ^3^H-thymidine incorporation three days later. iDC: immature DC: monocytes treated for 7 days with GM-CSF + IL-4. DC: iDC stimulated with LPS for 24 h. (B) Maturation of classical iDCs. Immature DCs were treated as indicated for 3 days. Expression of costimulatory molecules (grey histograms) was determined by flow cytometry. Isotype control (open histograms). Numbers in the graphs indicate the percentages of positive cells. *p<0.05. Data are representative of three independent experiments.

Classical DCs derived from Balb/C mice induced a strong proliferation of T cells in a mixed lymphocyte reaction (MLR). CD137-Fc-treated monocytes were devoid of this activity and also the addition of LPS during the last 24 h could not confer a T cell stimulatory activity. Fc-treated control monocytes also did not induce T cell proliferation (Figure 3A).

Human immature classical DCs that were generated from CD34^+^ hematopoietic progenitor cells or peripheral blood by cytokine stimulation could be matured by CD137 ligand agonists to inflammatory DCs [16]–[18]. After having found that in murine monocytes CD137 ligand signaling does not induce differentiation to inflammatory DCs we wondered whether it would at least induce maturation of immature classical DCs.

Total bone marrow cells were differentiated to immature DCs by a 7 day culture with GM-CSF + IL-4 and were then matured with either LPS or CD137 protein. Maturation by LPS strongly increased expression of CD40 (8.9 vs 33.7% positive cells) and CD86 (21.0 vs 60.1% positive cells) whereas the Fc control protein or CD137-Fc had no effect. Expression of CD80 was not changed by any of the three treatments (Figure 3B). These data indicate that CD137 ligand signaling not only fails to induce DC differentiation in murine monocytes but also maturation of immature DCs.

### The role of cell attachment in monocyte activation

In order to determine whether CD137-induced activation of monocytes was a consequence of recombinant CD137-Fc protein binding to CD137 ligand and thereby inducing monocyte attachment we compared the effects of CD137-Fc protein to the effects of two related proteins. Like CD137, TNF receptor I (TNFRI) and CD134 (OX40) are members of the TNFR family, and TNFRI-Fc and CD134-Fc protein are constructed in a similar manner and resemble in structure the CD137-Fc protein.

**Figure 4:**
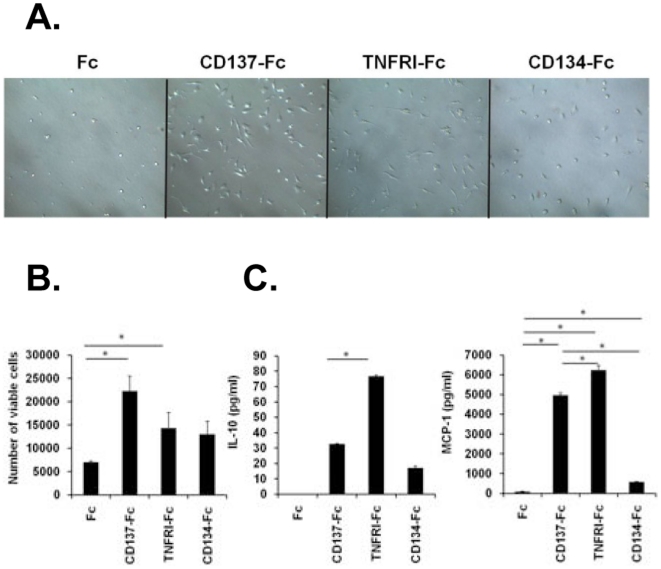
Comparison of CD137-Fc, TNFRI-Fc and CD134-Fc. Monocytes were cultured under indicated conditions for 7 days. (A) Morphological changes were documented by photography at 20x magnification. (B) Numbers of viable cells were determined by flow cytometry using Sphero Accucount Blank Particles. (C) The concentrations of IL-10 and MCP-1 in the supernatants were measured by ELISA. N.D: Not detectable. *p<0.05. Data are representative of three independent experiments.

TNFRI-Fc induced attachment and morphological changes in murine monocytes that were similar to the ones induced by CD137-Fc, while CD134-Fc was inactive (Figure 4A). The numbers of live cells were elevated in all three treatment groups with CD137-Fc having the most potent effect (Figure B). TNFRI-Fc induced the highest secretion of IL-10 and MCP-1. CD134-Fc only induced low levels of MCP-1 and no IL-10 (Figure 4C).

These data demonstrate the qualitatively different effects of the three recombinant receptor fusionproteins on monocyte attachment, viability and activity, and indicate that the activities of recombinant CD137-Fc protein are due to CD137 ligand signaling, and are not merely a consequence of CD137-induced monocyte attachment.

### Analysis of alternative myeloid cell populations and CD137 ligand agonists

**Figure 5:**
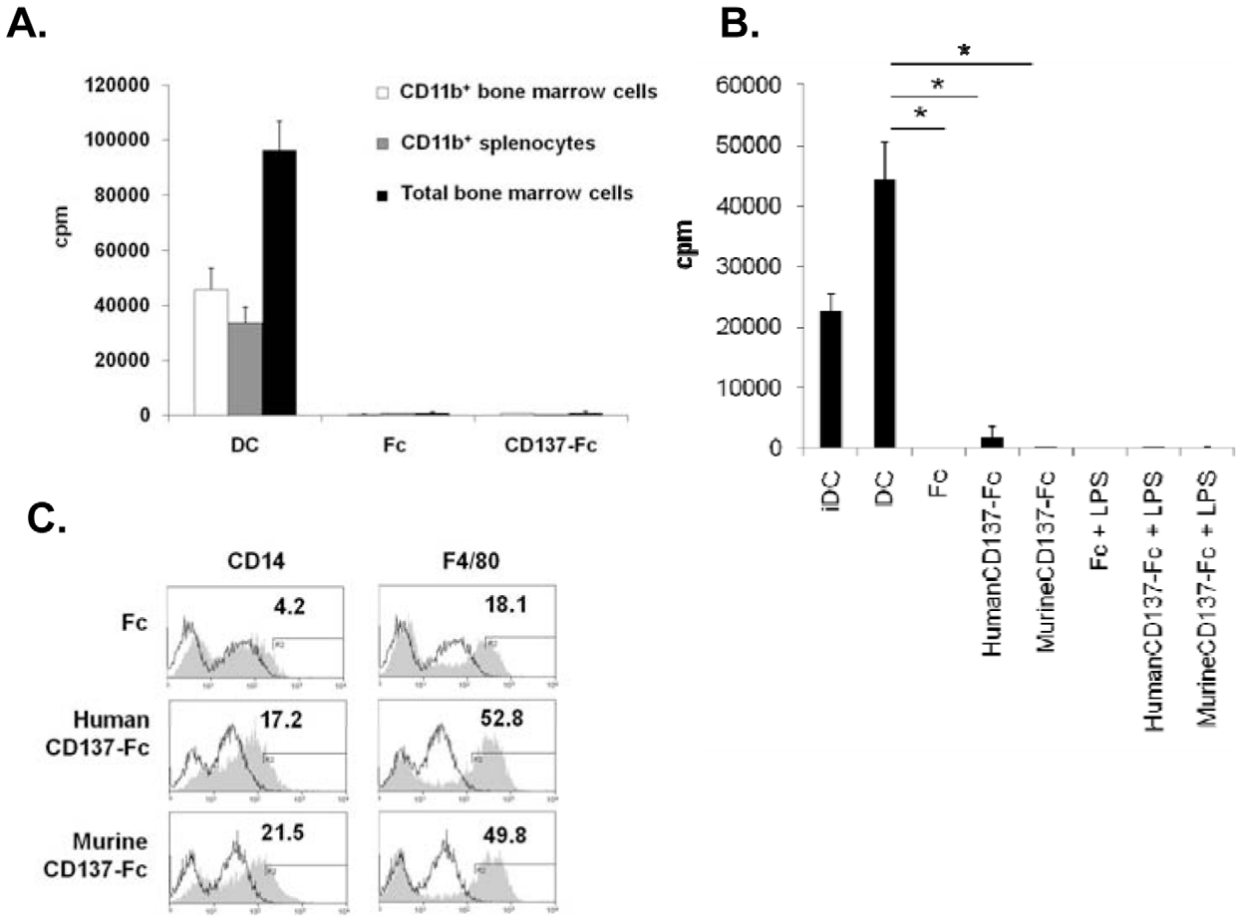
Alternative myeloid cell populations and CD137 ligand agonists. (A) Source of cells: Balb/C CD11b^+^ cells from bone marrow and spleen and total bone marrow cells were treated with immobilized Fc or CD137-Fc protein. (B, C) Source of CD137 protein: CD11b^+^, Ly6G^−^ monocytes were treated with immobilized Fc or human or murine CD137-Fc protein. Cells differentiated to immature and mature classical DCs were included as controls. (B) After 7 days the cells were used as stimulators in a MLR with C57/Bl6 T cells at a ratio of 1∶10. Proliferation was determined by ^3^H-thymidine incorporation at day 3 of coculture. (C) Expression of CD14 and F4/80 was determined at day 7 of the culture. iDC: immature DC. *p<0.05. Data are representative of three independent experiments.

The above data point to a clear species difference between human and murine monocytes in regards to the effects of CD137 ligand signaling. However, a concern was that the observed differences may be due to the different cell populations. Since it is not feasible to isolate large numbers of peripheral murine monocytes CD11b^+^, Ly6G^−^ bone marrow cells were used instead since they are regarded as the murine equivalent of human peripheral monocytes. To further confirm the obtained results and to ensure that the obtained data are representative of murine monocytic cells and not just to CD11b^+^, Ly6G^−^ bone marrow cells we treated total bone marrow cells, CD11b^+^ bone marrow cells and CD11b^+^ spleen cells with CD137-Fc protein. An allogenic MLR showed that upon treatment with CD137-Fc protein neither bone marrow nor splenic CD11b^+^ cells differentiated to stimulatory DCs (Figure 5A).

For all of the above experiments we had used human CD137-Fc protein. Though human CD137 protein had been shown to activate murine leukocytes [12], [26] we stimulated CD11b^+^, Ly6G^−^ monocytes with human and murine CD137-Fc protein. There was no difference in the phenotype of the cells as neither protein was able to induce differentiation to stimulatory DCs as reflected by their inability to secrete IL-12 (not shown) and by their inability to induce T cell proliferation in an allogeneic MLR (Figure 5B). Also, the expression of the macrophage markers CD14 and F4/80 was highly comparable on monocytes that were treated with human or murine CD137-Fc protein (Figure 5C). This rules out that the difference we observed between human and murine monocytes to CD137 stimulation was due to a mismatched protein.

## Discussion

It was recently reported that CD137 ligand signaling induces differentiation of peripheral human monocytes to inflammatory DCs, and that these CD137L-DCs are more potent than classical DCs that were generated by GM-CSF + IL-4 and matured by LPS + IFN-γ. DCs hold great potential for immunotherapy but currently patient response rate is still low, and induced immune responses are often too weak [27], [28]. More potent DCs would be expected to enhance the efficacy of immunotherapy. The generation of the murine equivalent of the human CD137L-DCs promised to allow extending the characterization of CD137L-DCs from in vitro experiments to murine models and thereby obtaining more relevant in vivo data on the potency of CD137L-DCs.

Based on the reports with human CD137L-DCs we expected CD137 ligand signaling to induce differentiation of murine monocytes to inflammatory DCs. Indeed, the phenotypical response, i.e. increased attachment, morphological changes and induction of proliferation were identical between monocytes from the two species. But surprisingly, CD137 treatment failed to generate inflammatory DCs from murine monocytes.

Addition of LPS as a maturation factor did not convert the monocytes to inflammatory DCs but rather enhanced their IL-10 secretion while inducing no IL-12. Therefore, it has to be concluded that a species difference exists between murine and human monocytes in their differentiation response to CD137 ligand signaling. While human monocytes differentiate to inflammatory DCs murine monocytes do not. It is at present entirely unknown what the biological reason for this species difference may be.

This species difference becomes also evident in the effects of CD137 ligand signaling on immature classical DCs that were generated by GM-CSF + IL-4. Exposure of human immature, classical DCs to CD137 protein or CD137-expressing cells induces maturation as evidenced by higher expression of costimulatory molecules and IL-12, stronger migratory activity and a higher T cell stimulatory capacity [16]–[18]. In contrast, CD137 ligand signaling did not induce costimulatory molecule and IL-12 expression in murine immature, classical DCs.

This species difference may have its molecular basis in the low conservation of human and murine CD137 ligand. While the amino acid sequence identity between other human and murine members of the TNF family range between 70 and 80%, it is only 36% for CD137 ligand [29]. Therefore, it seems plausible that different signaling pathways and accordingly different biological effects are initiated by human and murine CD137 ligand signaling. Nevertheless, this species difference was unexpected since CD137 ligand signaling has the same effect on human and murine hematopoietic progenitor cells and induces macrophage differentiation in both species [26], [30]. However, species differences between man and mouse have already been described for the CD137 receptor/ligand system. While CD137 signaling delivers potent costimulatory signals to both human and murine T cells it affects NK cells differently in the two species. Crosslinking of CD137 enhances the activity of murine NK cells [31] while it inhibits cytotoxicity and IFN-γ release of human NK cells [32].

Due to the species difference of CD137 ligand signaling on human and murine monocytes future in vivo studies to characterize CD137L-DCs cannot be conducted in mice. The further characterization of CD137L-DCs has to rely on in vitro experiments with human cells or on mice with a reconstituted human immune system or possibly on primates.
